# Subcutaneous versus Transvenous Implantable Cardioverter Defibrillator in Patients with End-Stage Renal Disease Requiring Dialysis: Extended Long-Term Retrospective Multicenter Follow-Up

**DOI:** 10.3390/jpm14080870

**Published:** 2024-08-17

**Authors:** Fabian Schiedat, Benjamin Meuterodt, Joachim Winter, Magnus Prull, Assem Aweimer, Michael Gotzmann, Stephen O’Connor, Christian Perings, Thomas Lawo, Ibrahim El-Battrawy, Christoph Hanefeld, Johannes Korth, Andreas Mügge, Axel Kloppe

**Affiliations:** 1 Department of Cardiology and Angiology, Marienhospital Gelsenkirchen, Academic Hospital of the Ruhr University Bochum, 45886 Gelsenkirchen, Germany; 2 Department of Cardiology and Angiology, University Hospital Bergmannsheil Bochum of the Ruhr-University Bochum, 44789 Bochum, Germanyandreas.muegge@ruhr-uni-bochum.de (A.M.); 3 Department of Cardiology, Electrophysiology, Pneumology and Intensive Care Medicine, St. Marien-Hospital Luenen, Academic Hospital of the University Muenster, 44534 Lünen, Germany; 4 Department of Cardiovascular Surgery, Heinrich-Heine University Hospital, 40225 Duesseldorf, Germany; 5 Department of Cardiology, Augusta Hospital Bochum, Academic Hospital of the University Duisburg Essen, 44791 Bochum, Germany; 6 Department of Cardiology, Katholische Kliniken Bochum of the Ruhr University Bochum, 44791 Bochnum, Germany; michael.gotzmann@ruhr-uni-bochum.de (M.G.);; 7 Department of Biomedical Engineering, City, University of London, London WC1E 7HU, UK; 8 Department of Cardiology, Elisabeth Hospital Recklinghausen, 45661 Recklinghausen, Germany; 9 Department of Molecular and Experimental Cardiology, Institut für Forschung und Lehre (IFL), Ruhr-University Bochum, 44791 Bochum, Germany; 10 Department of Nephrology, University Hospital Essen, University of Duisburg-Essen, 45147 Essen, Germany

**Keywords:** sudden cardiac death, implantable cardiac defibrillator, S-ICD, TV-ICD, CIED infection, end-stage renal disease, hemodialysis

## Abstract

Background: Implantable cardioverter defibrillators (ICD) prevent sudden cardiac death (SCD). Patients with end-stage renal disease (ESRD) requiring dialysis are at a very high risk of infection from cardiac implantable electronic device (CIED) implantation as well as mortality. In the present study, we compared the long-term complications and outcomes between subcutaneous ICD (S-ICD) and transvenous ICD (TV-ICD) recipients. Methods: In this retrospective analysis, we analyzed a total of 43 patients with ESRD requiring dialysis who received either a prophylactic S-ICD (26 patients) or a single right ventricular lead TV-ICD (17 patients) at seven experienced centers in Germany. Follow-up was performed bi-annually, at the end of which the data concerning comorbidities and, if applicable, reason for death were checked and confirmed with patients’ general practitioner, nephrologist and cardiologist. Results: The median follow up duration was 95.6 months (range 42.8–126.3 months). Baseline characteristics were without noteworthy significant differences between groups. During follow-up (FU), there were significantly more device-associated infections (HR 8.72, 95% confidence interval (CI), 1.18 to 12.85, *p* < 0.05) and device-associated hospitalizations (HR 10.20, 95% CI 1.22 to 84.61, *p* < 0.001), as well as a higher cardiovascular mortality (HR 9.17, 95% CI 1.12 to 8.33, *p* < 0.05), in the TV-ICD group. The number of patients requiring hospitalization for any reason was significantly higher in the TV-ICD group (HR 2.59, 95% CI 1.12 to 6.41, *p* < 0.05). There was no significant difference in overall mortality (HR 1.92, 95% CI 0.96 to 6.15, *p* = 0.274). Conclusions: Our data suggest that, in this extended follow-up in seriously compromised renal patients on dialysis, the S-ICD patients have statistically fewer device infections and hospitalizations as well as lower cardiac mortality compared with the TV-ICD cohort.

## 1. Introduction

Implantable cardioverter defibrillators (ICD) are recommended to reduce the risk of sudden cardiac death (SCD) in primary and secondary prevention indications in patients with expectation of good quality survival of more than one year [1]. In patients who have recovered from hemodynamically unstable ventricular arrhythmia without a reversible cause, an ICD is recommended for secondary prevention. In patients with New York Heart Association (NYHA) class II or III, symptomatic heart failure with reduced left-ventricular ejection fraction (HFrEF), left ventricular ejection fraction (LV-EF) ≤ 35% despite ≥ 3 months of optimal medical treatment, an ICD is recommended for primary prevention [1,2].

Where cardiac resynchronization, bradycardia, or anti-tachycardia pacing (ATP) is not required, either a subcutaneous implantable cardioverter defibrillators (S-ICD) or a conventional transvenous implantable cardioverters defibrillators with a single right ventricular lead (TV-ICD), is recommended for both primary and secondary prevention [1]. TV-ICD is associated with higher lead related complications during long-term follow-up compared with S-ICD, whilst demonstrating comparable safety and efficacy [3]. TV-ICD in patients requiring hemodialysis (HD) is associated with peri- and postoperative complications related to venous access and infection [4]. Transvenous lead extractions (TLE) are associated with high morbidity and mortality, especially in device-related infections [5]. As the vasculature is preserved with S-ICD implantations, systemic infections are less frequent and interventions are rare and less invasive [6,7]. Long-term data on the outcome of patients requiring cardiac implantable electronic devices (CIED) and suffering from end-stage renal disease (ESRD) requiring HD are very limited. The aim of the present study was to investigate the long-term outcome of patients who received either an intermuscularly implanted S-ICD or a TV-ICD with a single right ventricular lead in a cohort of patients suffering from ESRD requiring HD.

## 2. Methods

This study is a retrospective analysis of patients who received either an intermuscularly placed S-ICD or TV-ICD, who did not require pacing or ATP and suffered from concomitant ESRD requiring HD. Patients were recruited from seven experienced centers (>100 ICD implantations per year and center) in Germany (University Hospital Bergmannsheil Bochum, Marienhospital Gelsenkirchen, University Hospital Katholische Kliniken St. Josef Bochum, University Hospital Duesseldorf, Marien-Hospital Luenen, Augusta Hospital Bochum and Elisabeth Hospital Recklinghausen) with the same standard approach for device implantation. The devices were implanted between 2009 and 2018. Informed consent was obtained. The study protocol conforms to the ethical guidelines of the 1964 Declaration of Helsinki and its later amendments. It was approved by the ethics committee of the Ruhr University Bochum (Register 22-7593-BR). All participating patients had indications for an ICD following the guidelines and a good quality life-expectancy > 1 year, despite ESRD requiring HD [1,2]. Medical history was obtained from the hospital records.

### 2.1. Implantation Procedure

Implantation procedure was performed under local anesthesia with deep sedation. All patients received intravenous antibiotic prophylaxis according to the local protocols prior to the procedure. The decision to implant either a TV-ICD or S-ICD was done according to operators’ discretion. The generator of the S-ICD (Emblem S-ICD, Model 209, Boston Scientific, Marlborough, MA, USA or SQ-RX 1010, Cameron Health, San Clemente, CA, USA) was implanted intermuscularly between the anterior surface of serratus anterior and the posterior surface of the latissimus dorsi, in an intermuscular space between the mid and posterior axillary lines over the left fifth or sixth rib [3,8,9]. The S-ICD electrode was implanted via a two or three incision technique and sutures were applied at all incision sites. Induced arrhythmia conversion testing was performed for all S-ICD implantations, and the electrode was repositioned in case of non-conversion until successful conversion.

For TV-ICD implantation, the lead was placed via the axillary, cephalic or subclavian vein and the pulse generator was placed in a pre-pectoral, sub-fascial or sub-muscular position. Sutures were applied at the sleeve and the generator. Induced arrhythmia conversion testing was not performed in TV-ICD implant procedures. Implant procedure-related complications were categorized according to severity [10].

### 2.2. Patient Follow-Up

All patients were examined before discharge for final device interrogation and wound control and for the S-ICD, all the vectors were tested. Follow-up (FU) was performed initially after 3 months and afterwards bi-annually in the outpatient department. Device interrogation, complications, hospitalizations and any other events were documented according to the definitions proposed by the European Heart Rhythm Association (EHRA) guidelines [11]. If a patient did not attend FU, patient, general practitioner, nephrologist and/or cardiologist were contacted to document if the patient was alive, in which case all possible complications and hospitalizations were documented and the patient was asked to attend future FUs. Alternatively, cause of death was ascertained.

### 2.3. Data Collection

Data have been analyzed retrospectively but were collected continuously according to hospitals’ standard and checked afterwards with hospital information system.

### 2.4. Statistical Analysis

Statistical analyses were performed on Mac OS using IBM SPSS Statistics version 28.0.0. Categorial variables were stated as frequencies and percentages for normal distribution or median and interquartile range for non-normal distribution. Continuous variables were expressed as mean ± standard deviation. Linear regression was used to assess descriptive statistics and baseline characteristics. Chi-quadrat test was used for normally distributed, non-normally distributed and binary data with linear trends if required. Kaplan–Meier analyses were performed for time to primary endpoints, time free of hospitalization and death, and compared with a log-rank test. Hazard ratios were estimated with Cox multivariate regression analysis.

## 3. Results

### 3.1. Patient Population and Implant Procedure

**Figure 1:**
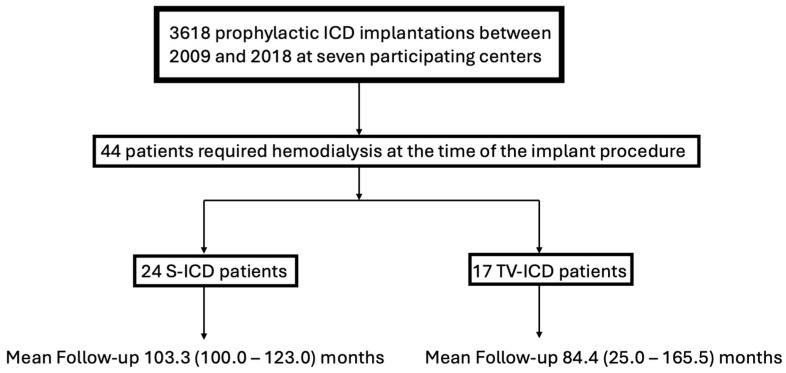
Flow-chart presenting patient inclusion and follow-up.

A total of 44 patients with end-stage renal disease requiring HD, a life expectancy greater than one year and indication for ICD implantation were initially included in this study. One patient in the TV-ICD group was excluded from all analyses due to an indication for cardiac resynchronization therapy (CRT) upgrade during FU, due to progressive heart failure and left bundle branch block. A total of 43 patients were ultimately included for analysis. All procedures were performed between 2009 and 2018 by seven experienced operators, one at each center. During the same period, a total of 3618 prophylactic S-ICD implantations and TV-ICDs with single right ventricular lead were performed at the seven participating centers. S-ICD was implanted intermuscularly in 26 patients. Left-sided TV-ICD was implanted in 17 patients with a single-coil right ventricular lead. A flow-chart presenting the inclusion is illustrated by Figure 1. All patients required HD for ESRD at the time of the implant procedure and during the whole FU.

Baseline demographics in each group were not significantly different, except for more S-ICD patients with a history of failed renal transplantation. For baseline medication, more patients in the TV-ICD group received diuretics, betablockers and antiarrhythmic medication. More patients in the S-ICD group received acetylsalicylic acid (ASS), dual antiplatelet medication (DAPT) and immunosuppression medication.

All of the S-ICD implantation attempts were successful with a 15 joule safety margin being established from induced arrhythmia conversion testing, with three patients (11.5%) requiring an electrode position change and retest. Implant duration was significantly longer in the S-ICD group (71.4 (55.4–87.4) vs. 54.8 (37.5–72.5), *p* < 0.001). Minor perioperative complications were significantly lower in the S-ICD group (3.8%, vs. 29.4%, *p* < 0.05) with no major peri- or postoperative complications occurring. In the TV-ICD group, one (5.8%) patient had a pneumothorax after the procedure, three (17.6%) patients suffered from a pocket hematoma requiring revision and one (5.8%) patient required a lead revision for dislodgment. In the S-ICD group, one (3.8%) patient required a pocket revision for hematoma.

A comparison of baseline and implant procedure characteristics is listed in Table 1.

### 3.2. Follow-Up

An overview of all FU variables collected is listed in Table 2. Mean FU duration was 103.3 (100.0–123.0) months and 84.4 (25.0–165.5) for the S-ICD groups and TV-ICD respectively. However, no patient required a change of system for a pacing indication in the S-ICD cohort. No patient in the TV-ICD cohort required more than 5% ventricular pacing.

**Figure 2:**
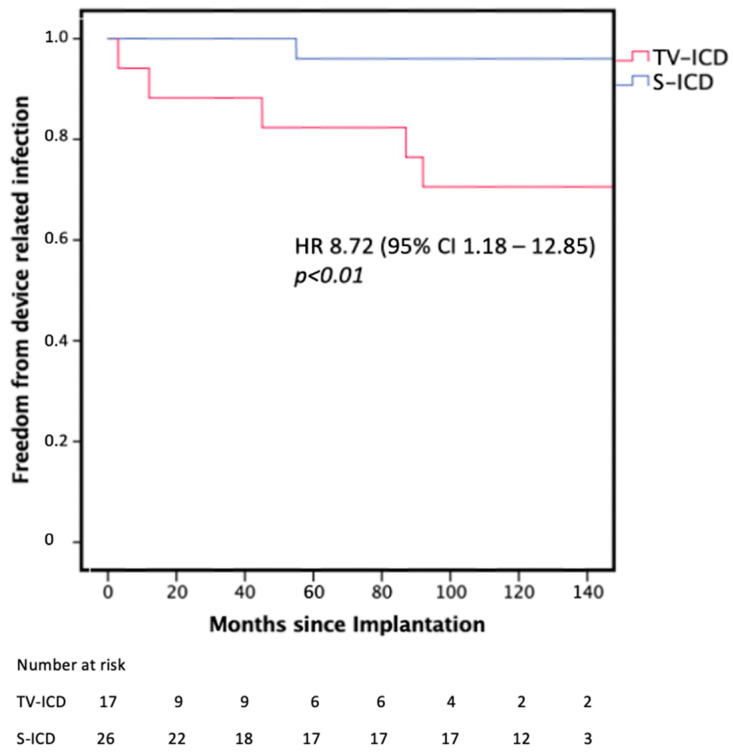
Kaplan–Meier graph illustrating the freedom from device-associated infections requiring device removal. TV-ICD = Transvenous ICD; S-ICD = Subcutaneous ICD.

During FU, device-related infections (HR 8.72, 95% confidence interval (CI), 1.18 to 12.85, *p* < 0.05) and device-associated hospitalizations (HR 10.20, 95% confidence interval (CI), 1.22 to 84.61, *p* < 0.001) occurred significantly more often in the TV-ICD group, as illustrated in Figure 2. All five (29.4%) infections in the TV-ICD group required TLE and system removal, while the one (3.8%) S-ICD-related pocket infection was handled conservatively and successfully with antibiotic therapy without the need for surgical intervention. Device-related infections in the TV-ICD group were an isolated pocket infection in one patient, a pocket infection with bacteremia in two patients and lead endocarditis in two patients.

**Figure 3:**
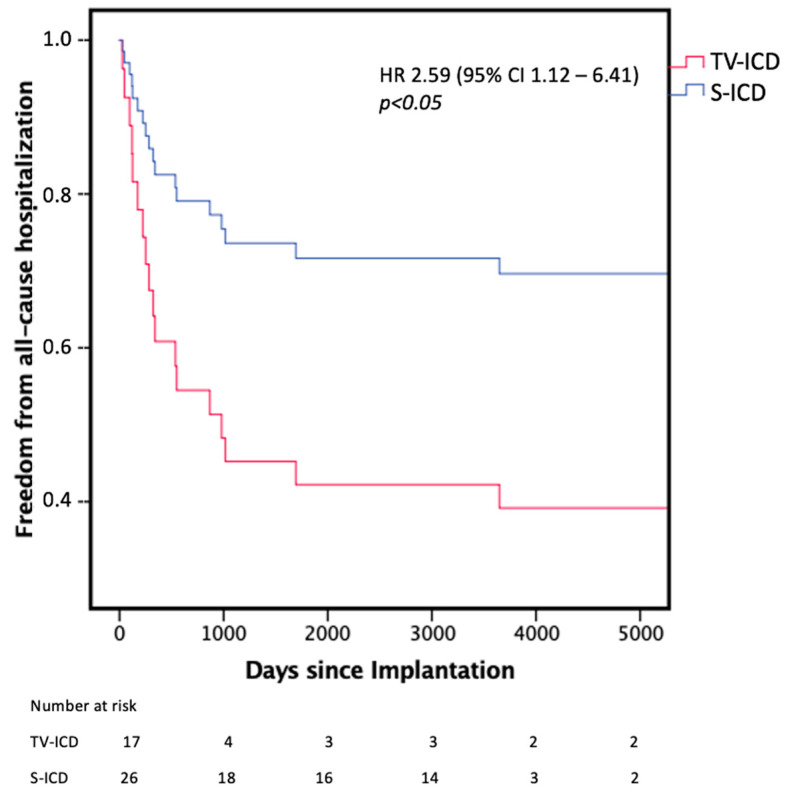
Kaplan–Meier graph illustrating the freedom from all-cause hospitalization. TV-ICD = Transvenous ICD; S-ICD = Subcutaneous ICD.

**Figure 4:**
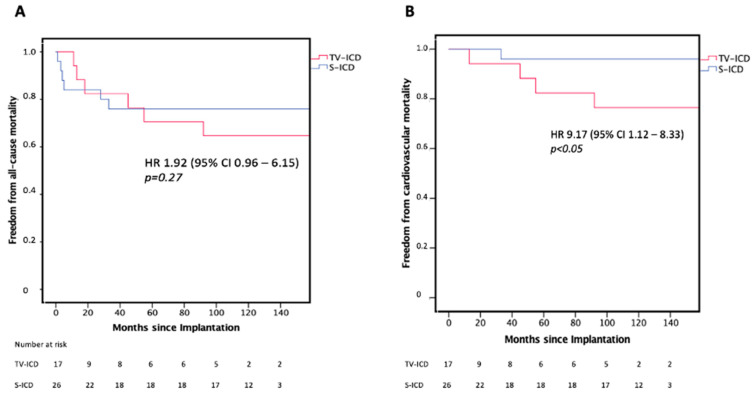
Kaplan–Meier plot for freedom from all-cause mortality (**A**) and freedom of cardiovascular mortality (**B**) since procedure. TV-ICD = Transvenous ICD; S-ICD = Subcutaneous ICD.

In the TV-ICD group cardiovascular mortality (HR 9.17, 95% CI 1.12 to 8.33, *p* < 0.05), all-cause hospitalization (HR 2.59, 95% CI 1.12 to 6.41, *p* < 0.05), total all-cause hospitalizations per patient (3.4 ± 2.9 vs. 0.8 ± 1.8, *p* < 0.05), number of cardiac hospitalizations per patient (1.3 ± 2.4 vs. 0.9 ± 0.9, *p* < 0.05) and duration of all-cause hospitalization (62.0 ± 22.6 days vs. 24.0 ± 18.6 days, *p* < 0.05) were significantly higher in the TV-ICD group compared with the S-ICD group. All-cause mortality was not significantly different (HR 1.92, 95% CI, 0.96 to 6.15, *p* = 0.274). Difference in all-cause hospitalization is illustrated in Figure 3. Freedom from all-cause mortality and freedom of cardiovascular mortality since procedure is illustrated in Figure 4.

Patients experiencing episodes of ventricular arrhythmia, receiving appropriate and inappropriate shocks, were not significantly different between groups. In the S-ICD group, two patients received inappropriate shocks due to oversensing. T-wave oversensing occurred in one patient because of low amplitude signals and atrial fibrillation with low amplitude signals in the other. Both were managed by a change in sensing vectors in the outpatient clinic without further sequelae. In the TV-ICD group, no inappropriate shocks occurred.

## 4. Discussion

We have investigated whether there is a difference in the long-term outcome of patients receiving either an S-ICD or TV-ICD while suffering from ESRD requiring HD. Patients with ESRD exhibit an markedly elevated risk for coronary artery disease, heart failure, arrhythmias and sudden cardiac death [12]. The baseline demographics in both groups from our cohort is in line with large data sets published for HD patients and their reported prevalence for cardiovascular diseases [13]. According to guidelines, there are no specific recommendations for ICD therapy in patients with ESRD, even though patients with progressive CKD (chronic kidney disease) have been excluded from most studies demonstrating a reduced mortality for ICD patients compared with patients receiving drug therapy [1,14]. Patients with progressive CKD (GFR ≤ 29 mL/min/1.73 m^2^) have been excluded from the S-ICD Investigational Device Exemption Trial as well [15]. In the S-ICD post-approval study, 220 (13.4%) of 1637 patients were undergoing HD at the time of implant procedure [16]. Comparative outcomes between S-ICD and TV-ICD in this highly compromised group of patients, however, have not been previously published.

According to analyses of a nationwide database of 40,075 ICD implants, [17], ESRD is associated with a significantly higher proportion of intraoperative complications, especially bleeding, vascular injury and in-hospital mortality. In our cohort, no vascular complications or major procedure-related complications occurred, regardless of the device type. Minor intra- or postoperative complications occurred significantly more often in patients receiving a TV-ICD. The complication rates in our study are in line with previously reported data by Ayoub et al. [17] in TV-ICD recipients.

Patients receiving DAPT have longer device implant times, reportedly due to bleeding and the association of DAPT with a higher incidence of postoperative hematoma [18]. Even though significantly more patients in our S-ICD group received DAPT, less minor procedure-related complications occurred. A reason could be longer hemostasis as operators knew of the risk in patients with DAPT, which could explain the longer S-ICD procedure times. Another reason for longer procedure time could be induced arrhythmia conversion testing in the S-ICD cohort with the need to reposition the S-ICD electrode in three patients.

Cardiovascular rather than ESRD associated mortality is the leading cause for death in patients with advanced stage 4 and 5 CKD [12]. A meta-analysis by Tonelli et al. demonstrated an exponential increase in absolute risk for death with decreasing kidney function [19]. In patients with advanced CKD, especially in those requiring HD, SCD accounts for more than two-thirds of mortality [12,20]. Wan et al. demonstrated in a cohort of 75 HD patients, who experienced a sudden cardiac arrest, that 78.6% of these were due to unstable ventricular arrhythmia rather than asystole [21]. Patients undergoing HD were a significant univariable predictor for appropriate and inappropriate shocks in a 5-year FU of the S-ICD post-approval study [22]. There was no significant difference between our two groups with respect to the number of patients experiencing ventricular arrhythmias during FU. The number of appropriate and inappropriate shocks was comparable to a large prospective non-inferiority comparison between TV-ICD and S-ICD, the latter with a slightly shorter FU [6]. Our data showed a comparable number of appropriate shocks delivered compared with patients on HD in the post-approval S-ICD study [16]. Bradycardias have been described in advanced CKD as well, however without any relation to a reduced LV-EF [23]. In the post-approval S-ICD study, Gold et al. reported in a population of 1643 patients, a rate of only 1.6% of patients requiring a change of system for pacing during FU of up to 5 years [22]. For the same cohort, El-Chami et al. reported one death due to asystole while suffering from myocardial infarction according to autopsy for a subgroup consisting of 220 HD patients [16]. There were no patients who needed a change of system for bradycardia in our S-ICD cohort and there were no patients with a ventricular pacing above 5% in the TV-ICD group. It therefore appears to be safe to implant an S-ICD in patients requiring HD, even though bradycardias have been described in this population.

Device lifetime infection rate is around 2–3% for conventional ICD systems according to the Danish ICD registry in a general population [24]. There is an association for catheter-related bacteria and TV-ICD infections [25] as patients often receive HD through a central venous access. CKD is an independent risk factor for device infections [26]. There was no difference in all-cause mortality in a prospective trial comparing TV-ICD vs. best usual care in patients receiving HD [27]. Device-associated complications, however, occurred during a mean FU of 6.8 years in twenty-five (31.3%) of eighty patients with TLE and whole system removal due to bacteremia in four (5%) of all eighty patients [27]. Guha et al. reported a CIED infection rate of 8% in TV-ICD ESRD patients with only TLE and device removal improving outcome moderately [4]. Weiss et al. reported on 293 S-ICD recipients without a strict intermuscular implantation strategy and a CIED-associated infection in 18 (6.1%) patients [15]. The rate of patients requiring system removal for CIED-associated infection however was very low, four (1.3%) patients overall and 4/18 (22.2%) patients of all S-ICD related infections. Thirteen (72.2%) of the remaining fourteen patients with infection were being handled conservatively with antibiotics, and one (5.6%) underwent sternal wound revision [15]. The authors described a reduction of CIED infection during the course of the study and attributed this to standardization and optimization of the implantation techniques [15]. Device-associated infections occurred in our cohort significantly more often in the TV-ICD group, with all cases requiring TLE and ICD system removal, whereas conservative therapy with antibiotics was effective in the single S-ICD patient. In the post-approval S-ICD study, there was no difference in device-related infections between patients requiring HD and the rest of the cohort [16]. Boersma et al. performed a retrospective analysis of 75 patients who received an S-ICD after TLE of an infected TV-ICD system. They reported only one case of device-associated infection in this cohort of patients at high-risk for recurrent infection during a FU of nearly two years [28]. The vasculature is preserved in S-ICD implantations. It seems possible that the S-ICD with no leads on or in the heart has a lower incidence of device-related infections and endocarditis. The S-ICD therefore prevents a common cause for further significant complications including hospitalization, complex TLE procedures and mortality. In addition, the feasibility of creating a arteriovenous fistula, as the gold standard of vascular access in patients requiring HD, can be complicated due to a reported rate between 11 and 36% of venous occlusion or central vein stenosis in patients with TV-ICDs or presence of a CIED device on one site, leaving only the other site for HD vascular access [29].

Device-related complications and CIED-related infections are common causes for hospitalization in patients with CIED. Device and lead related complications occur significantly more often in TV-ICD recipients compared with S-ICD recipients, according to a large meta-analysis by Fong et al. [3]. This low complication rate is supported by the post-approval S-ICD study, showing that patients with HD in this real-world cohort had a higher rate of appropriate and inappropriate shocks delivered by the S-ICD, but no increase in device-associated complications [16]. In our cohort, CIED-related complications occurred significantly more often in the TV-ICD cohort. These CIED-associated complications and infections were the main triggers for a higher rate of hospitalizations in the TV-ICD group. Non-cardiac hospitalizations were not significantly different between groups. In line with the higher rate of cardiovascular hospitalizations, we saw a higher cardiovascular mortality in our TV-ICD cohort. Even though antiarrhythmic medication was higher in the TV-ICD group at baseline, ventricular or atrial arrhythmic episodes were not the cause during FU. Overall mortality, however, was also not significantly different between groups. Overall mortality adjusted for FU duration in our cohort is comparable to other large trials involving patients requiring HD and ICD therapy [16]. There was no difference in all-cause mortality in our study compared with a prospective trial comparing TV-ICD vs. best usual care in patients requiring HD [27]. However, the mortality rate in our cohort was lower. An explanation could be that only patients with the expectation of good quality survival of more than one year have been implanted.

### Limitations

Despite many centers participating, the sample size remains small. Patients requiring HD, expecting a of good quality survival of more than one year and having an indication for an ICD are rare. This is in line with data reported from a total of 22,443 ICD implant procedures in Germany in 2019, of which only 326 (1.45%) patients required dialysis for ESRD [30]. In our participating centers, 1.22% of all ICD procedures required dialysis for ESRD, therefore illustrating a real-world cohort.

This study represents the only comparative analysis of TV-ICD and S-ICD recipients while requiring HD. For a more valid conclusion, a prospective, randomized trial would be beneficial. In patients with presumed non-cardiac deaths, no device interrogation has been performed to confirm this.

## 5. Conclusions

This long-term follow up of seriously compromised hemodialysis patients, at risk of sudden cardiac death and requiring a CIED has shown that the S-ICD system is safe and effective. No device-related complications or infections compromised survival in patients with an S-ICD. These findings support further prospective studies comparing S-ICD and TV-ICD in patients with renal insufficiency, especially in those requiring hemodialysis.

## Figures and Tables

**Table 1:** Baseline and procedure characteristics.

	TV-ICD (n = 17)	S-ICD (n = 26)	*p*-Value
Age (years)	65.1 (54.5–73.0)	64.9 (56.5–79.0)	0.967, ns
BMI (kg/m^2^)	27.0 (24.1–30.2)	24.8 (22.0–28.3)	0.177, ns
Female gender, n (%)	3 (17.6)	5 (19.2)	0.892, ns
Primary prophylactic, n (%)	10 (58.8)	18 (69.2)	0.386, ns
Anemia, n (%)	10 (58.8)	5 (19.2)	0.128, ns
Hyperparathyroidism, n (%)	5 (29.4)	3 (11.5)	0.148, ns
Previous renal transplant, n (%)	0	4 (15.4)	0.087, ns
Ischemic cardiomyopathy, n (%)	10 (58.8)	18 (69.2)	0.108, ns
NYHA class	2.3 ± 1.3	2.5 ± 0.5	0.381, ns
Coronary artery disease, n (%)	12 (70.6)	21 (80.8)	0.301, ns
Myocardial infarction, n (%)	11 (64.7)	11 (42.3)	0.195, ns
History of heart surgery, n (%)	6 (35.3)	8 (30.8)	0.831, ns
Atrial arrhythmia, n (%)	8 (47.1)	10 (38.5)	0.387, ns
Arterial hypertension, n (%)	13 (76.5)	20 (76.9)	0.791, ns
Diabetes, n (%)	10 (58.8)	10 (38.4)	0.269, ns
Hyperlipoproteinemia, n (%)	11 (64.7)	16 (61.5)	0.964, ns
Chronic obstructive lung disease, n (%)	1 (5.9)	1 (3.8)	0.785, ns
Stroke of ischemic and non-ischemic etiology, n (%)	1 (5.9)	4 (15.4)	0.365, ns
Liver disease, n (%)	3 (17.6)	0	0.083, ns
Peripheral artery disease, n (%)	3 (17.6)	1 (3.8)	0.146, ns
History of vascular surgery, n (%)	1 (5.9)	1 (3.8)	0.785, ns
Potassium (mmol/L)	4.8 ± 0.5	4.9 ± 0.6	0.515, ns
Hemoglobin (g/dL)	11.4 ± 1.4	11.6 ± 2.3	0.811, ns
CRP (mg/dL)	2.9 ± 2.9	2.1 ± 2.0	0.286, ns
Betablocker, n (%)	9 (52.9)	5 (19.2)	<0.05
ACE inhibitor/ARB, n (%)	4 (23.5)	2 (11.5)	0.216, ns
MRA, n (%)	1 (5.9)	0	0.332, ns
Diuretics, n (%)	8 (47.1)	4 (15.4)	<0.05
Anti-arrhythmic medication, n (%)	6 (35.3)	3 (11.5)	<0.05
Cardiac glycosides, n (%)	1 (5.9)	1 (0.4)	0.785, ns
ASS, n (%)	7 (41.2)	22 (84.6)	<0.05
DAPT, n (%)	3 (17.6)	12 (46.2)	<0.05
OAK, n (%)	7 (41.2)	6 (23.1)	0.642, ns
Corticosteroid, n (%)	2 (11.8)	4 (15.4)	0.496, ns
Immunosuppression medication, n (%)	0	4 (15.4)	<0.05
Insulin, n (%)	7 (41.2)	5 (19.2)	0.143, ns
LVEF (%)	31.3 (26.3–43.8)	27.9 (25.0–30.5)	0.243, ns
Implant duration (min)	54.8 (37.5–72.5)	71.4 (55.4–87.4)	<0.05
Minor perioperative complications, n (%)	5 (29.4)	1 (3.8)	<0.05
Perioperative vascular complications	0	0	ns

TV-ICD = Transvenous ICD; S-ICD = Subcutaneous ICD; BMI = Body mass index; NYHA = New York Heart Association; CRP = C-Reactive protein; ACE = Angiotensin converter enzyme; ARB = Angiotensin II receptor antagonists; MRA = Mineralocorticoid receptor antagonist; ASS = Acetylsalicylic acid; DAPT = Dual anti-platelet therapy; OAK = Oral anti-coagulant therapy; LVEF = Left ventricular ejection fraction.

**Table 2:** Follow-up.

	TV-ICD (n = 17)	S-ICD (n = 26)	Hazard Ratio (95% CI)	*p*
Duration follow-up, months	84.4 (25.0–165.5)	103.3 (100.0–123.0)		0.305, ns
Infection requiring whole system removal, n (%)	5 (29.4)	0		<0.005
Device-related infection, n (%)	5 (29.4)	1 (3.8)	8.72 (1.18–12.85)	<0.01
Time to first device infection, (months)	47.8 (7.5–89.5)	-		
Lead associated complication	2 (11.7)	0		0.188, ns
Overall mortality, n (%)	6 (35.3)	6 (23.1)	1.92 (0.96–6.15)	0.274, ns
Cardiovascular mortality, n (%)	4 (23.5)	1 (3.8)	9.17 (1.12–8.33)	<0.05
Patients experiencing ventricular arrhythmia	3 (17.6)	2 (7.7)		0.355, ns
Mean number ventricular arrhythmia episodes	0.7 ± 1.8	0.1 ± 0.3		0.150, ns
Average ATP	0.2 ± 0.5	0		ns
Patients receiving a shock, n (%)	2 (11.7)	4 (15.4)		0.388, ns
Patients receiving appropriate shocks	2 (11.7)	2 (7.7)		0.605, ns
Patients receiving inappropriate shocks, n (%)	0	2 (7.7)		0.242, ns
Patients receiving more than one shock	0	0		
Hospitalization rate, n (%)	11 (64.7)	8 (30.8)	2.59 (1.12–6.41)	<0.05
Mean number hospitalizations per patients	3.4 ± 2.9	0.8 ± 1.8		<0.05
Patients with cardiac hospitalization, n (%)	7 (41.2)	3 (11.5)	5.99 (1.24–28.9)	<0.05
Mean cardiac hospitalizations	1.3 ± 2.4	0.2 ± 0.9		<0.05
Patients with non-cardiac hospitalization, n (%)	7 (41.2)	4 (15.4)	1.43 (0.86-1.73)	0.158, ns
Number of non-cardiac hospitalizations	0.8 ± 1.5	0.4 ± 1.3		0.377, ns
Patients with device hospitalization, n (%)	7 (41.2)	1 (3.8)	10.2 (1.22-84.61)	<0.001
Overall duration hospitalization (days)	62.0 ± 22.6	24.0 ± 18.6		<0.05

TV-ICD = Transvenous ICD; S-ICD = Subcutaneous ICD; ATP = Anti-tachycardia pacing.

## Data Availability

All data can be made available by Fabian Schiedat upon request.

## Abbreviations

BMI	Body mass index
CIED	Cardiac implantable electronic device
CKD	Chronic kidney disease
CRP	C-reactive proteins
CRT	Cardiac resynchronization therapy
ESRD	End-stage renal disease
GFR	Glomerular filtration rate
HD	Hemodialysis
HF	Heart failure
HFrEF	Heart failure with reduced ejection fraction
ICD	Implantable cardioverter defibrillator
LV-EF	Left ventricular ejection fraction
Min	Minutes
NYHA	New York Heart Association
SCD	Sudden cardiac death
S-ICD	Subcutaneous implantable cardioverter defibrillator
TLE	Transvenous lead extraction
TV-ICD	Transvenous implantable cardioverter defibrillator
